# Research on coal mine longwall face gas state analysis and safety warning strategy based on multi-sensor forecasting models

**DOI:** 10.1038/s41598-024-64181-7

**Published:** 2024-06-14

**Authors:** Haoqian Chang, Xiangrui Meng, Xiangqian Wang, Zuxiang Hu

**Affiliations:** School of Economics and Management, Anhui University of Science & Technology, Huainan, 232000 China

**Keywords:** Mining safety, Mining management engineering, Time series analysis, Warning strategy, Data mining, Machine learning, Engineering

## Abstract

Intelligent computing is transforming safety inspection methods and response strategies in coal mines. Due to the significant safety hazards associated with mining excavation, this study proposes a multi-source data based predictive model for assessing gas risk and implementing countermeasures. By examining the patterns of gas dispersion at the longwall face, utilizing both temporal and spatial correlation, a predictive model is crafted that incorporates safety thresholds for gas concentrations, four-level early warning method and response strategy are devised by integrating weighted predictive confidence with these correlations. Initially tested using a public dataset from Poland, this method was later verified in coal mine in China. This paper discusses the validity and correlation of multi-source monitoring data in temporal and spatial correlation and proposes a risk warning mechanism based on it, which can be applied not only for safety warning but also for regulatory management.

## Introduction

Gas concentration is a pivotal parameter in assessing excavation safety within coal mines, almost all mining operations are associated with gas concentration, and it is also the primary factor to coal mines accidents^1^. Predictions and monitoring of large amounts of gas data are feasible^2,3^, but the important bridging between abstract complex physical systems and their users’ needs is missing. That is, there is a need for the conversion of these predictions to a level explainable by metrics that reflect the true working scenarios and importantly, a decision-making level by human users^4^.

The formidable operational environment of coal longwall faces (the place where coal is cut from the coal seam either manually or mechanically), characterised by semi-enclosed tunnels, a plethora of sensors, diverse equipment, extreme temperature and pressure conditions^5^, and the ever-present risk of gas-related accidents^6^, adds substantial complexity to the assessment of overall safety. Furthermore, the complexity of geological conditions^7^ and structures across different longwall faces complicates the assessment of the general safety status.

**Figure 1:**
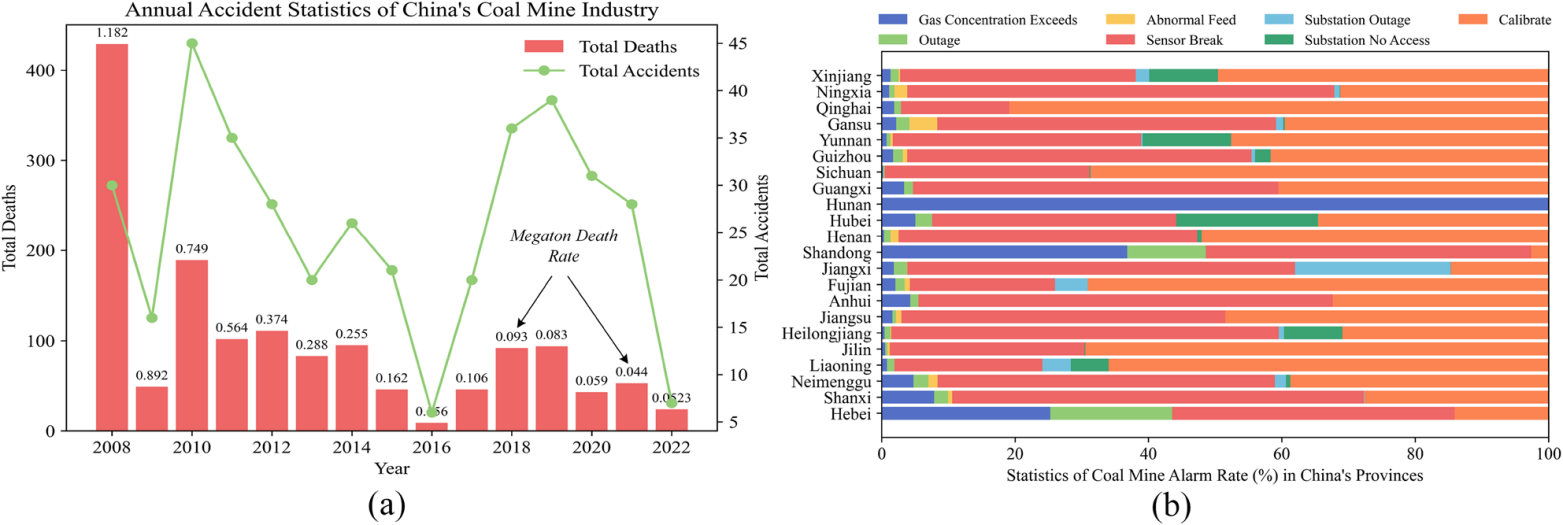
(**a**) Recent trends in China’s coal mine accident rates and mortality per million tons^12^. (**b**) Key causes of gas-related underground sensor alarms.

Due to the rapid development of Industry 4.0^8^, traditional manual monitoring and management methods in coal longwall face are not sufficient^9^. Although accidents and megaton mortality rates in China’s coal mines have shown a downward trend^10,11^, the overall alarm rate remains high (see Fig. 1). Where Fig. 1a shows the total number of accidents and casualties in China’s coal mines from 2008 to 2022, as well as the annual megaton coal mining mortality rate. However, whilst accidents are most distressing, it is important to note also alarms that have not led to accidents (yet). Figure 1b, displays the number of alarms (in the form of percentage) related to gas in underground coal mines in each province of China from 2020 to 2022. Investigating these warnings remains labor-intensive; fortunately, they have not escalated into serious accidents. However, it is crucial that they are accurately categorized and systematically reviewed to prevent future incidents.

Hence, there is a growing demand for advanced early warning strategies and countermeasures to ensure coal mining face safety. The primary research areas related to this include: gas concentration prediction through data analysis^13^, accident prevention and early warnings, especially for incidents like gas explosions and coal and gas herniations^14^, and digital twin modelling^15^, whom focusing on the coordination of coal mining equipment^16^. Other areas include safety monitoring and risk warning related to gas concentration^17^, roof pressure prediction^18^, multifactor coupling and decoupling of the coal mining face^19^, etc. Despite the diversity of these studies, the analysis based on gas concentration remains the predominant research direction. This is because it offers the most direct insight^1,9^ into the underground environment and serves as the primary criterion for warning mechanisms.

The study of early safety warning strategies^20^ for coal mine longwall faces is an important part of promoting coal mine safety. Traditionally, mine safety management uses production thresholds to trigger alarms at certain concentrations. by classifying coal mines into high, medium and low types for underground gas emissions and coal production. Current applications^21^ primarily focus on safety and production status display, as well as warnings for exceeding predefined limits. Based on the influencing factors related to gas concentration as variables. Ding et al.^22^ proposed a gas concentration prediction method based on partial least squares regression. However, as digitalization and automation advance, there is an increasing need for accurate and early risk warnings but has several challenges. First, monitoring data is the result of a combination of events, including gas absorption^23^ and decomposition at the coal wall, coal mining operations, and coal transportation. Moreover, as the longwall face expands, the pattern of this combination will also change. Secondly, there are multiple sensors at various locations collectively monitoring the status of the gas concentration throughout the longwall face. This means that the values from these sensors reflect the direction of movement of the gas in the longwall face and the trend of the concentration, which contains both geographical and time lag information. However, this type of information is difficult to interpret directly by forecasting. A coal mine longwall face presents a semi-enclosed environment^24^, characterised by high temperatures and pressures, exhaust gases, humidity and darkness, among other complexities. High-precision sensors, such as methane and carbon monoxide, operating in this environment are extremely prone to malfunction, damages by flying stones, as well as malicious manmade masking—resulting in nulls, abnormal values^25,26^. This raises the challenge of how to handle and recognise these anomalies, which is important for the creation and evaluation of the overall safety warning strategy.

Gas concentration as highly time-dependent data, make deep learning based intelligent algorithms^27,28^ demonstrated their capability to process and predict large volumes of multi-source data in various aspects of coal mine safety management^9^, gas concentration^29^. But simply relying on conventional metrics, such as accuracy, or learning test loss, to define gas trends, is insufficient, as these losses do not reflect the spatial correlation of gas concentrations, i.e., the sorption effect of the gas as it drifts from position A to position B in the longwall face, nor do they explain the effect of time lag on the prediction results. Ultimately, they do not provide managers with the intuitive risk rating they need^30^.

To address these challenges and to evaluate the real-time working conditions of the coal mine longwall face, early safety warning strategies are proposed and adopted in underground coal mine safety management^31,32^. Current methodologies predominantly depend on manual observation and heuristic approaches. In contrast, the implementation of big data models utilizing deep learning^33^ allows for comprehensive monitoring and evaluation of the entire work environment. This advancement not only enhances managerial decision-making but also mitigates human error. To development of an interpretable safety warning strategy relies on two main aspects: (i) Multivariate-based time-series prediction of a large amount of gas monitoring data to decipher high-dimensional coupling relationships; (ii) identification and analysis of dynamic multi-source data variation patterns across both time and space. Thus, we propose a safety warning strategy that utilizes multi-sensor monitoring data, incorporating it into a four level risk assessment system with an evolution mechanism. The strategy is validated on a publicly available dataset from Poland and a coal mine in Anhui, China.

## Methodologies

### Coal mine data description

**Figure 2:**
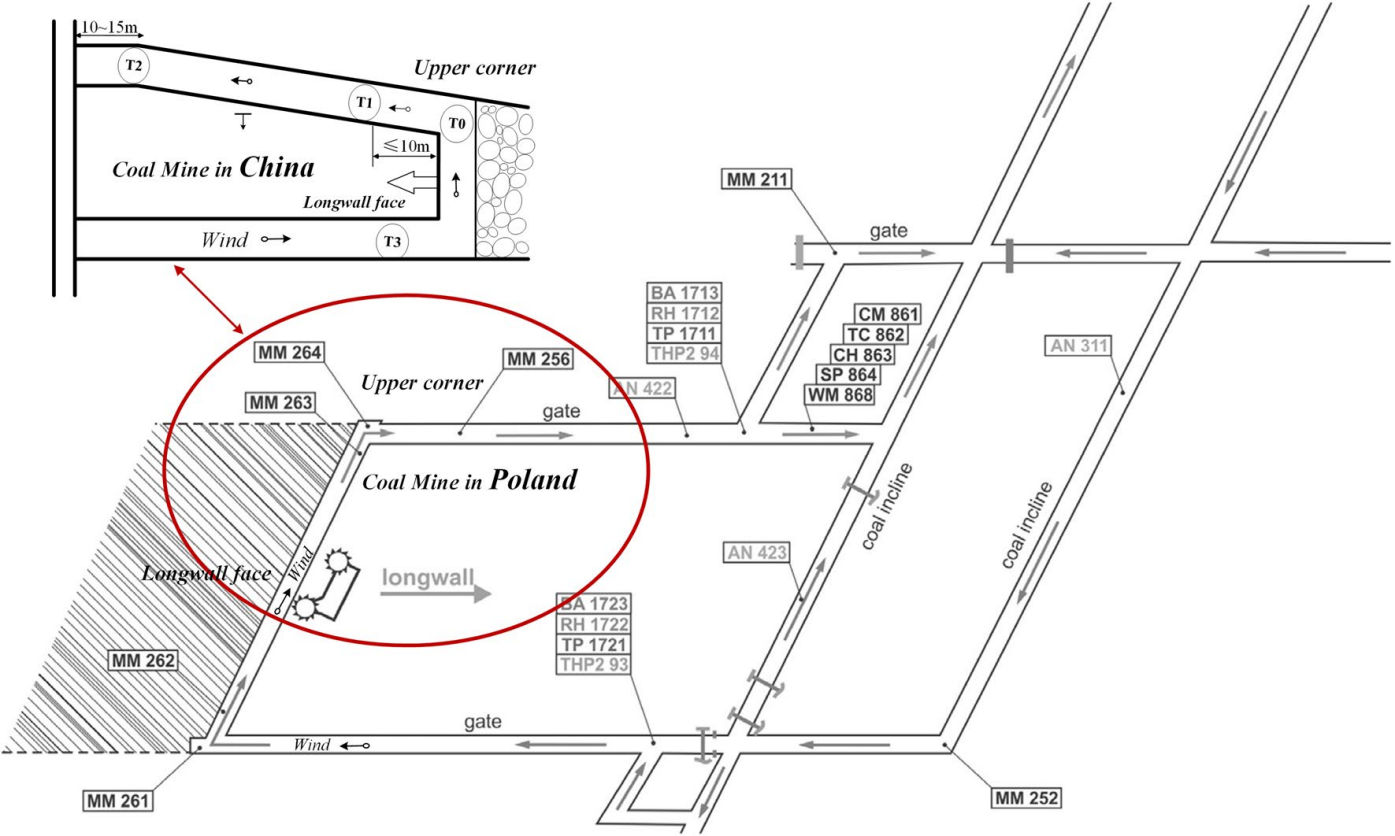
Comparison of coal mine gas sensor locations in two coal faces in China and Poland (sensors start with T in China and MM in Poland).

The experimental data for this study comprises two main sources: a publicly available dataset from coal mine in Poland^26,34^ and the data we collected from a coal mine in China;the dataset from the coal mine in Poland was obtained from the Upper Silesian Coal Basin, consisting of time-sensitive readings from March 2, 2014, to June 16, 2014, with a total of 9,199,930 data instances, each detailed with timestamps and measurements. Meanwhile, the dataset from the coal mine in China was acquired using gas sensors in the W3211 working face at Qianyingzi Mine, Suzhou City, Anhui Province, during the period from May 1, 2021, to July 20, 2021, accumulating a total of 3,407,328 data points. Both datasets have been preprocessed, with timestamps standardized to the minute level. An overview of the main gas sensors for the two locations can be seen in Fig. 2. The upper corner represents a critical gas monitoring point on the coal mining face. This location is significant due to intense activities such as digging, blasting, coal dropping, and transportation. Furthermore, this is an area bustling with employees and equipment, including coal miners, hydraulic supports, and scraper conveyors, among others.

The configuration of the longwall face support in Polish coal mines bears a resemblance to that utilized in Chinese coal mines, as shown in Fig. 2. Both exhibit a *U* shaped layout, facilitating airflow from beneath and directing it through the upper corners. This similarity warrants a comparative analysis of the two mining operations. However, the ventilation structure of a coal mining face is critical to the safety of the mine as it directly affects the control of gas and dust and the working environment of the miners. We concurrently analyse the spatio-temporal relationships of MM264 (upper corner), MM264, and MM256 in the Polish coal mine, and T0 (upper corner), T1, and T2 in the Chinese coal mine. To avoid redundancy and prevent confusion in our descriptions, we will hereafter refer to the longwall face using the nomenclature of the Chinese coal mining industry.

From the data presented in Table 1, we observe consistent patterns across both datasets. Both datasets utilise the “threshold risk judgement” methodology and are organised in a time-series format. Intrinsically, the datasets depict a spatial pattern over time. These datasets are segmented into distinct levels, according to the exceeded concentration value thresholds. Specific details regarding the preprocessing of these datasets are worth mentioning. Both datasets undergo several preprocessing steps, including padding and removal of invalid values. Additionally, due to the uneven data collection frequencies of the sensors, it is essential to synchronize the data across the same time period for consistency, in this study, we averaged the data sampled over a one-minute period.

**Table 1:** Sensor data from coal mines in Poland and China.

Point	Sensor	Alarm (%)	Warning (%)	Min	Max	Mean	Std.dev	Median	Lengths
Coal mine in Poland	MM263	$\ge 1.5$	$\ge 1.0$	− 2.0	30.0	0.248	0.197	0.2	9,199,931
	MM264	$\ge 1.5$	$\ge 1.0$	− 2.0	40.0	0.327	0.206	0.3	9,199,931
	MM256	$\ge 1.5$	$\ge 1.0$	0.0	30.0	0.433	0.204	0.4	9,199,931
Coal mine in China	T0	$\ge 1.5$	$\ge 1.0$	0.0	2.0	0.254	0.098	0.26	7,135,789
	T1	$\ge 1.5$	$\ge 1.0$	0.0	2.0	0.244	0.091	0.24	7,135,789
	T2	$\ge 1.5$	$\ge 1.0$	0.0	2.0	0.273	0.093	0.28	7,135,789

In contrast, the data from the Poland mine remains in its raw form, suggesting a need for preprocessing before undertaking any experimental analyses. The coal mine longwall face functions as an integrated environment. It encompasses a multitude of equipment sources, diverse monitoring data, and unpredictable coal rock conditions. Developing a safety warning strategy based on a multi-sensor model entails understanding intricate coupling relationships concealed amidst vast amounts of monitoring data.

**Figure 3:**
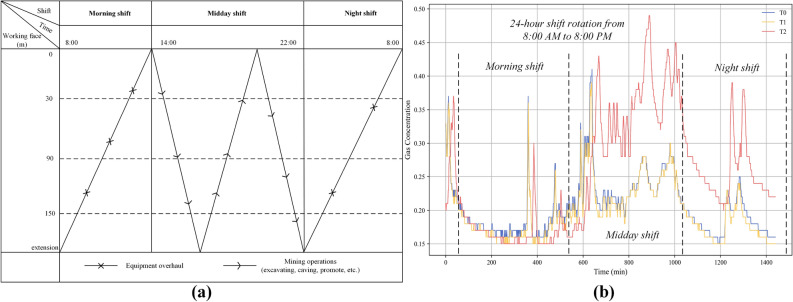
Coal mine operation roster in China and variation of gas concentration.

In China, coal mine operations generally run 24 h. a day, the intense coal mining operations primarily occur during daytime, with the remaining intervals designated for overhaul operations. During the mid-shift, which is the primary mining period, the gas concentration at the three locations spikes significantly, whereas it diminishes during the other two overhaul shifts, as depicted in Fig. 3. In Fig. 3a, the mining work shift schedule is plotted against the length of the longwall face (measured in meters) and the associated shift timings.

Four main shifts are observed: Morning, Midday, Night. Figure 3b illustrates the variations in gas concentrations as monitored by three distinct gas sensors: T0, T1, and T2, during the same shift times. As shown in Fig. 3, the horizontal axis represents time, segmented according to the primary work shifts— Morning, Midday, and Night. The vertical axis measures the levels of gas concentration. The data illustrates substantial diurnal fluctuations in gas concentration, characterized by distinct peaks and troughs. These peaks often correlate with periods of intense mining activity or specific processes within the mine, indicating a cyclical pattern to the gas emissions. Notably, there are significant increases in gas levels during the early hours of both the Morning and Midday shifts. A comprehensive analysis of both spatial and temporal variations enables a deeper understanding of the gas movement and diffusion patterns within the mine’s tunnels, encompassing both time and space dimensions.

### Temporal and spatial correlation

In coal mine longwall face, gas sensors are primarily categorised into different types, T0 (upper corner), which is no more than 800 mm from the roof plate, is the closest sensor to the coal mining position; T1: installed in the return airflow, within 10 m from the coal wall of the longwall face; T2: installed in the longwall face from the wind lane outlet to within 10–15 m, the distance between sensors is not explicit in the Polish dataset, so the same parameters are used. Each sensor gathers data, organised and stored in chronological sequences. These sequences reflect the state of coal mining operations and the corresponding conditions of the coal wall at different times. This can be expressed as $Senso{r_{0}} = \{ {x_1},{x_{2}},...,{x_i},...\}$, where ${x_i}$ denotes an observation at time *i* with a total length of points in time observed.

Cross-covariance functions for spatio-temporal (CCFST) multi-sensor data: Considering that gas in the longwall face drifts from sensor T0 to T1 due to wind flow, and as the workface continues to be excavated, the entire environment, as well as the location of the sensors changes. During the calculation process, time-space lagged values are generated, and a simple covariance calculation or Pearson’s correlation between the sensors would ignore potentially important patterns in the data. This spatial lag is crucial to understand how changes in gas concentration at one sensor location might influence or correlate with changes at another sensor location. The time lag helps in comprehending temporal correlations; it elucidates how fluctuations at a given moment might influence or correlate with subsequent changes, either at the same sensor or another.

The cross-covariance function for spatio-temporal data (CCFST) provides a measure of linear association between two time series (from different spatial locations) at different time points. It aids in deciphering how one sensor’s output fluctuates in relation to another’s across various spatial points and over time. Given sets of spatio-temporal sensor ${S_1}(s,t)$ and ${S_2}(s',t')$ , where *s* and *t* represent spatial location and time, respectively the CCFST can be define as Eq. (1):

1 $$\begin{aligned} {C_{{S_1}{S_2}}}(\mathbf{{\tau }},\lambda ) = E[({S_1}(s,t) - {\mu _1}(s,t))({S_2}(s + \mathbf{{\tau }},t + \lambda ) - {\mu _2}(s + \mathbf{{\tau }},t + \lambda ))], \end{aligned}$$

where $\tau$ and $\lambda$ are temporal and spatial lag, also denoted as $la{g_{temporal}}({\tau })$ and $la{g_{spatial}}(\lambda )$. *E* denotes the expectation. ${\mu _1}(s,t)$ and ${\mu _2}(s + \mathbf{{\tau }},t + \lambda )$ are the means of the processes at their respective locations and times.

Covariance measures the relationship between two datasets. If the datasets tend to increase and decrease together, the covariance is positive. If one set tends to increase when the other decreases, the covariance is negative. Each sensor, such as T0, T1, and T2, produces a time series of data over a consistent period. Even though each time series represents measurements from a different spatial location, the readings across these sensors at the same time can be viewed as a multi-dimensional data point. In our multi-sensor setup, the covariance matrix provides pairwise covariances for each sensor pair. The diagonal elements of the covariance matrix represent the variance of each sensor’s data, and the off-diagonal elements represent the covariance between the pairs of sensors. This allows us to analyse measurements from all the sensors.

### Prediction models

In the following the prediction models that have been applied in this work are described, together with the reason for using them.

Long short-term Memory (LSTM)^35^ networks have been developed to mitigate the issue of gradient vanishing encountered by standard Recurrent Neural Networks (RNNs) when processing long sequences. LSTM is set as the baseline model because of its mature architecture and its wide range of applications to facilitate comparison with more complex models. Incorporating the *gate* concept, LSTM comprise a *forgetgate*:${f_t}$, an *inputgate*:${i_t}$ and an *outputgate*:${o_t}$, each contributing to the model’s ability to retain or discard information through sequential data processing. The gates can be expressed as Eq. (2):

2 $$\begin{aligned} {{f}_{t}}=\sigma ({{W}_{f}}\cdot [{{h}_{t-1}},{{x}_{t}}]+{{b}_{f}}), \end{aligned}$$

where $\sigma$ is the sigmoid function of the forget gate ${{f}_{t}}$. ${{W}_{f}}$ and ${{b}_{f}}$ are the weights and biases, $h_{t-1}$ and ${x}_{t}$ are the hidden state of the previous time step and the input of the current time step.

3 $$\begin{aligned} {{i}_{t}}=\sigma ({{W}_{i}}\cdot [{{h}_{t-1}},{{x}_{t}}]+{{b}_{i}}) \end{aligned}$$

4 $$\begin{aligned} {{{{\tilde{C}}}}_{t}}=\tanh ({{W}_{C}}\cdot [{{h}_{t-1}},{{x}_{t}}]+{{b}_{C}}), \end{aligned}$$

where ${i}_{t}$ is the input gate, ${\tilde{C}}_{t}$ is the candidate unit status, and unit status update via Eq. (5).

5 $$\begin{aligned} {{C}_{t}}={{f}_{t}}_{{C}_{t-1}}+{{i}_{t}}_{{{{\tilde{C}}}}_{t}}. \end{aligned}$$

The output gate ${o}_{t}$ determines which information will be passed to the next loop:

6 $$\begin{aligned} {{o}_{t}}=\sigma ({{W}_{o}}\cdot [{{h}_{t-1}},{{x}_{t}}]+{{b}_{o}}) \end{aligned}$$

7 $$\begin{aligned} {{h}_{t}}={{o}_{t}}\tanh ({{C}_{t}}), \end{aligned}$$

where ${h}_{t}$ is the hidden state of the current time step, tanh() indicates the hyperbolic tangent function, ${W}_{o}$ and ${b}_{o}$ are the weights and biases of ${o}_{t}$.

Self-attention mechanism^36,37^ based models have become a cornerstone in NLP tasks, encompassing machine translation, text summarisation, and question-answering systems, among other sequence-based applications. The Transformer model stands out for its proficiency in handling long sequences, discerning intricate dependencies, and facilitating a high degree of parallel computation. The self-attention mechanism operates as Eq. (8):

8 $$\begin{aligned} Attention(Q,K,V) = softmax(\frac{{Q{K^T}}}{{\sqrt{{d_k}} }})V, \end{aligned}$$

where ${d_k}$ is the dimension of the key vector used to scale the dot product and *Q*, *K*, *V* are Query, Key and Value respectively. The multi-head attention mechanism as shown in Eqs. (9) and (10) enables the concurrent capture of various contextual relationships within a sequence. Coupled with positional encoding, this approach preserves the order of elements, ensuring that the model remains sensitive to the sequence’s syntactic structure.

9 $$\begin{aligned} MultiHead(Q,K,V) = Concat(hea{d_1},...,hea{d_h}){W^O}, \end{aligned}$$

10 $$\begin{aligned} hea{d_i} = Attention(QW_i^Q,KW_i^K,VW_i^V). \end{aligned}$$

### Evaluation metrics

To assess the predictive accuracy of various time series forecasting techniques, we utilise four distinct popular metrics. These include the mean squared error (MSE), root mean squared error (RMSE), mean absolute error (MAE), and mean absolute percentage error (MAPE). The MSE is a measure that captures the average squared differences between predicted and actual values. And RMSE, which is the square root of MSE, and MAE are absolute measures that quantify the prediction errors in their original scale. Conversely, MAPE offers a relative measure, providing an error percentage. All the metrics can be defined as:

11 $$\begin{aligned} MSE = \frac{1}{N}\sum \limits _{i = 1}^N {{{({y_i} - {{{{\hat{y}}}}_i})}^2}}, \end{aligned}$$

12 $$\begin{aligned} RMSE = \sqrt{\frac{1}{N}\sum \limits _{i = 1}^N {{{({y_i} - {{{{\hat{y}}}}_i})}^2}} }, \end{aligned}$$

13 $$\begin{aligned} MAE = \frac{1}{N}\sum \limits _{i = 1}^N | {y_i} - {{{{\hat{y}}}}_i}|,\end{aligned}$$

14 $$\begin{aligned} MAPE = \frac{1}{N}\sum \limits _{i = 1}^N {\frac{{|{y_i} - {{{{\hat{y}}}}_i}|}}{{{y_i}}}} \times 100\%, \end{aligned}$$

where the ${y_i}$ is the actual value, and ${{{{\hat{y}}}}_i}$ is predicted value.

## Safety warning strategy based on multi-sensor information

### Conceptual architecture

**Figure 4:**
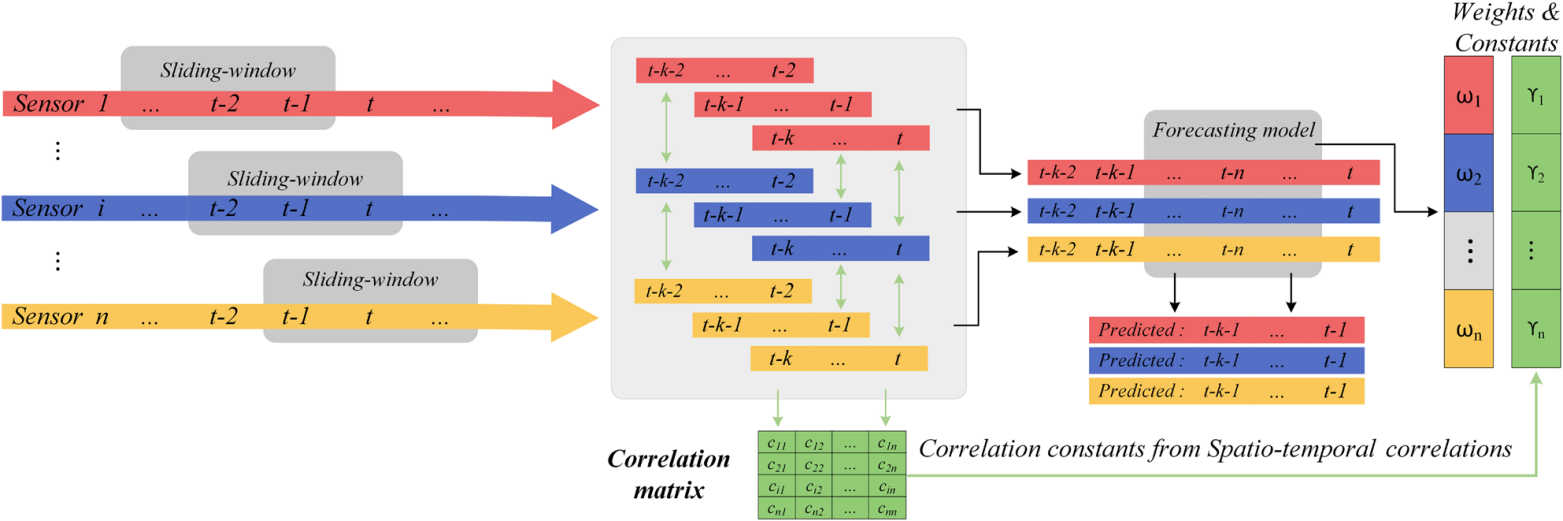
Multi-sensor data prediction and weight calculation.

To mitigate safety risks in the coal mining face and prevent emergency accidents, especially during mining operations, we propose a risk level warning system based on spatial–temporal correlations and multivariate time prediction models. This system encompasses risk determination, evolution mechanisms, and the implementation of corresponding safety measures. As depicted in Fig. 4, the multi-source sensor data prediction and weight calculation involve a sliding window approach which demonstrates a sliding-window methodology applied to data collected from multiple sensors, labelled from *Sensor*1 to *Sensorn*. This method involves continual data capture in temporal increments, processing the most recent data points while sliding past the older ones. Each sensor captures data at regular intervals, ensuring real-time monitoring.

Each sensor records data in temporal increments: the current data is labelled as *t*, while the two previous data points are labelled $t-1$ and $t-2$. Post the sliding-window processing, the data undergoes a correlation process with a time lag, essentially comparing a reading’s current value with its historical values. Understanding the temporal relationships of gas concentrations is crucial. This correlation provides insights into the behaviour and flow of the gas within the tunnel.

**Figure 5:**
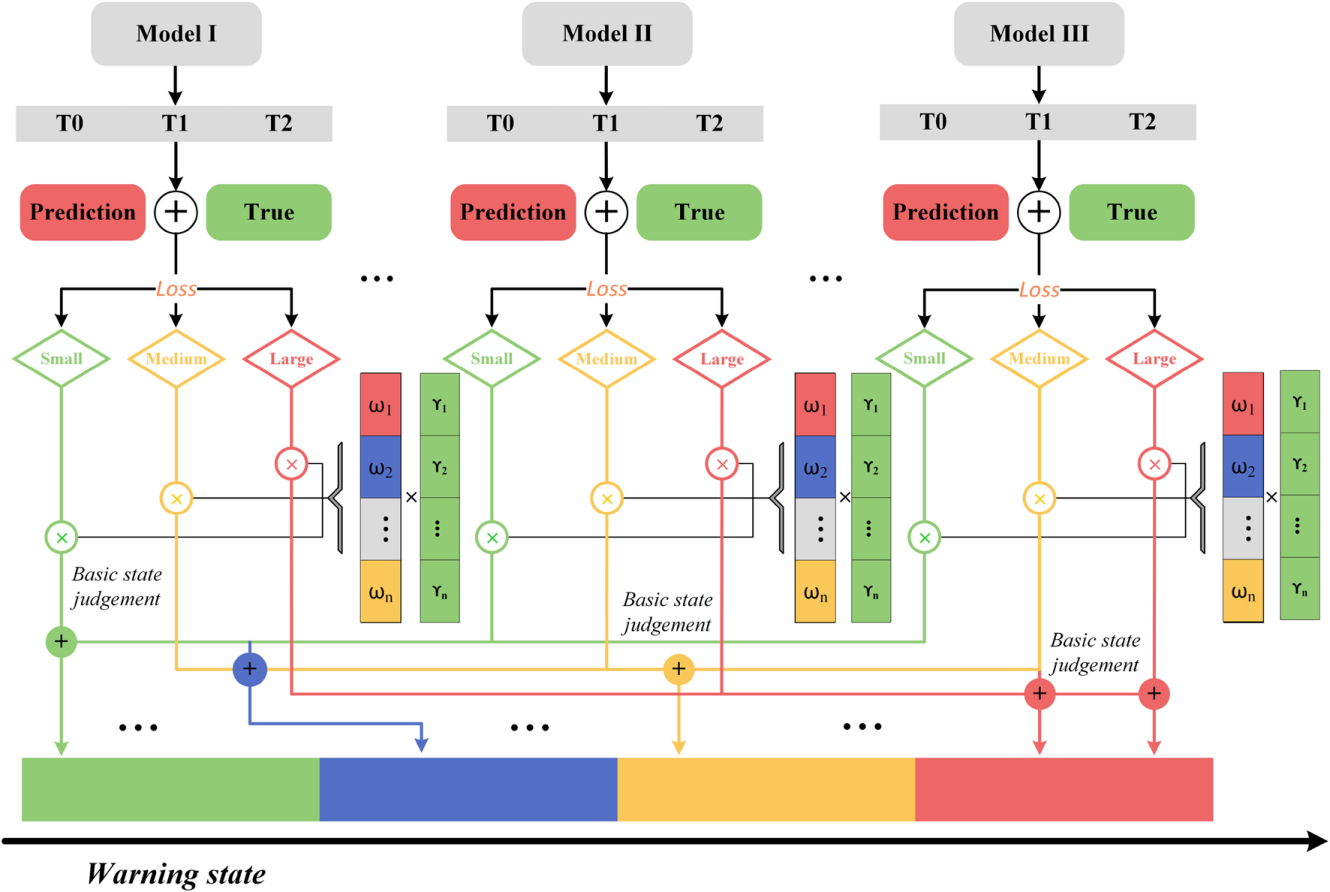
Weighted average based multi-sensor prediction models.

By sampling the multi-sensor monitoring values from Sensor1 to Sensor n through sliding windows and compute the correlation for the corresponding length time periods, calculating the correlation of a time series by segmentation can effectively identify individual patterns hidden in the overall correlation. At the same time, by controlling the size of the time window, not only the amount of computation can be alleviated, but also the time lag of the computation can be controlled, this time lag is particularly important in longwall face monitoring data, because most of the longwall faces in coal mines work according to a fixed scheduling pattern, i.e., two shifts and one inspection, as shown in the Fig. 3. The correlation matrix which contains the relationship between different data sources, and predictably, if one data source fails or changes drastically, the correlation matrix corresponding to the other data sources will also change, this provides constant weight CCFST for the risk discrimination mechanism, and variable weight which is losses from predictive models trained and predicted for each data source separately, by calculating the share of other data sources in this prediction (Fig. 5).

### Model architecture

Model loss influences early risk warning method which derived from a large number of calculations can be classified into three thresholds $\alpha ,\beta$ and $\gamma$ , are based on a large number of model predictions. When assessing the status of a single sensor, three primary states: *Red*, *Yellow*, and *Green*, are determined by correlating the current model loss with the score from the most recently assessed sensor. The specific thresholds $\alpha ,\beta$ and $\gamma$ can be adjusted based on the chosen model and metrics, allowing for tailored application. The comprehensive structure of this approach is illustrated in Fig. 5.

For each level there is a predicted value and ground truth. Loss thresholds are defined by calculating the loss between the predicted and actual values. A loss judgement can be defined as Eq. (15):

15 $$\begin{aligned} f(Basic\mathrm{{ }}State) = \left\{ {\begin{array}{*{20}{l}} {\mathrm{{Red}}}&{}{\mathrm{{if }}\left| {\omega - (1 - l)} \right| > \alpha }\\ {\mathrm{{Yellow}}}&{}{\mathrm{{if }}\beta \le \;\left| {\omega - (1 - l)} \right| \le \alpha }\\ {\mathrm{{Green}}}&{}{\mathrm{{if }}\gamma \le \left| {\omega - (1 - l)} \right| < \beta }. \end{array}} \right. \end{aligned}$$

**Figure 6:**
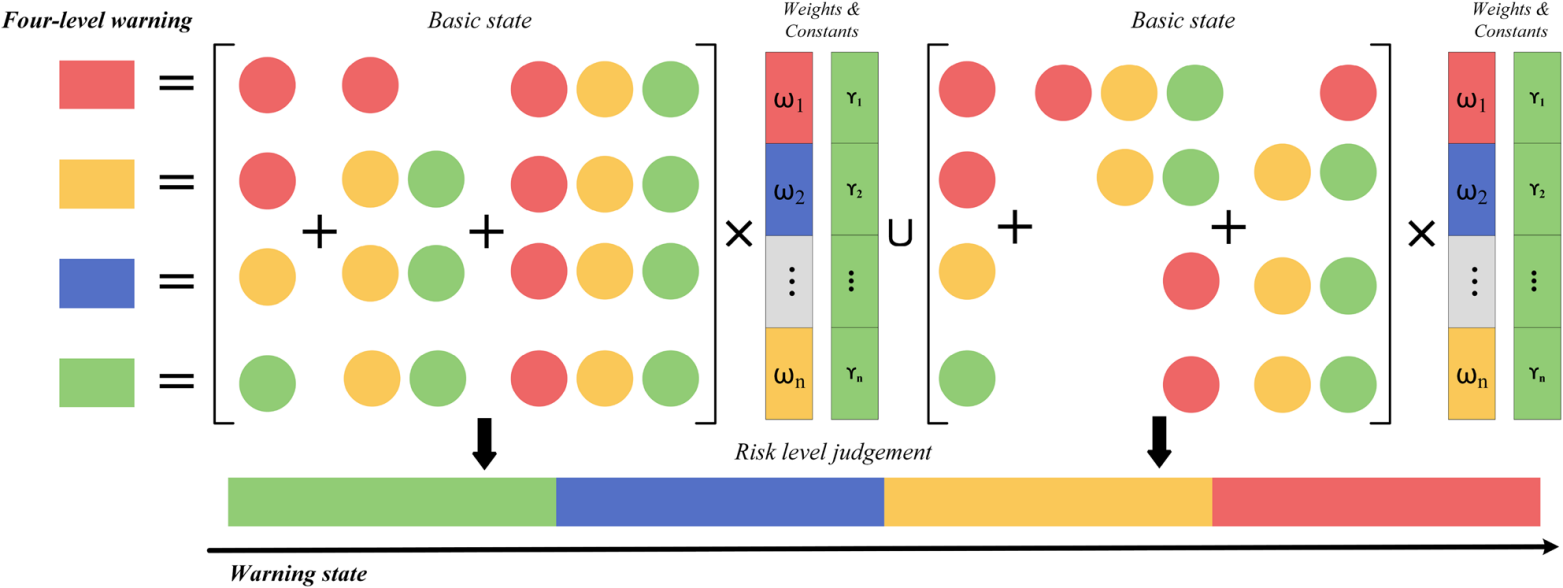
Four-level risk judgement and evolutionary mechanisms.

Each threshold has a corresponding weight, i.e., a prediction weight $\omega$ as well as a correlation weight $\gamma$. For example, when both $\omega$ and $\gamma$ rise and exceed the maximum threshold loss, defined as *Red* state, and when $\omega$ rises and $\gamma$ falls, defined as *Green* state. By using the sensors at different locations for state judgement, we can get a weighted risk judgement method as shown in Fig. 6 where the four risk levels are determined by basic state and their corresponding weights. Each level is indicated by a specific basic state, whereas multiple factors can collectively signify any of the states. This framework allows for a nuanced interpretation of risks based on the interplay and intensity of different factors.

### Risk evolution mechanisms and response plan

**Figure 7:**
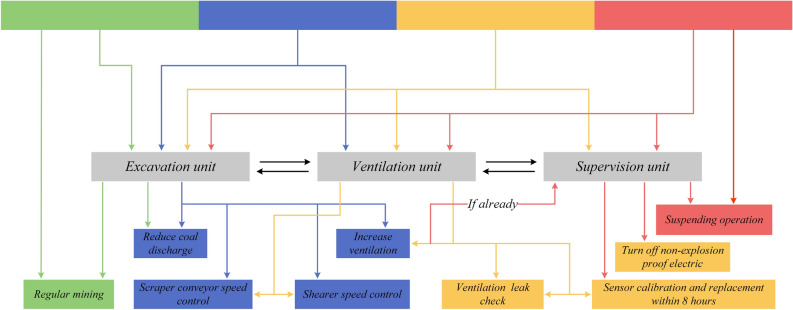
Workflow of the response plan.

We propose a four-level warning determination and multi-state representation as shown in Fig. 7. Combining multi-sensor data, which may represent a variety of sensor readings or states, with a series of weights and constants will result in the determination of four risk levels, each associated with a colour: red, yellow, blue and green. In combining the multi-state data and weights constants, a final risk judgement is derived, represented by a gradient from green to red, with increasing risk from left to right. By demonstrates that each risk class comprises a weighted combination of three distinct risk factors, which are made up of a spatio-temporal correlation matrix and modelled losses, where a structured multi-source sensor-based gas risk decision-making system around operational adjustments in a mining environment based on different states of warning conditions. At the top, ’warning conditions’ represent different levels of warning or risk, and each colour may indicate a different warning level, from low risk (green) to high risk (red). In relation to the actual mining process, actions are adjusted according to the warning status. And the safety response strategy is determined by the current working state together with the warning state, represented as Eq. (16):

16 $$\begin{aligned} Actio{n_{unit}} = f(Warnings\,State,Current\;Operational\;State). \end{aligned}$$

The safety strategy implemented within the mining operation is structured into three distinct parts, each designed to address specific aspects of the operational safety continuum. Firstly, the Excavation Unit is tasked with managing the core mining processes. Depending on the severity of the safety alerts received, this unit has the capability to adjust the coal discharge rate to mitigate risks effectively. Secondly, the Ventilation Unit is crucial for maintaining optimal air quality and gas concentrations within the mine. This unit’s responsibilities include actions such as increasing ventilation rates or adjusting the operational speed of the shearer to ensure a safe working environment. Lastly, the Supervision Unit acts as the oversight mechanism. It is empowered to implement robust safety measures, which may include halting operations or shutting down electric devices that could pose hazards under critical conditions.

The operational flowchart delineates a hierarchy of responses. These responses are designed to escalate from routine operational adjustments to more intensive, safety-focused interventions. This escalation is directly correlated with the progression from less severe to more severe warning states, ensuring a dynamic and responsive safety management system. Actions evolve from regular operational adjustments to intensive, safety-centric interventions as one transitions from the least to the most severe warning state.

## Experiments and results

### Spatio-temporal correlation of different coal mines

**Figure 8:**
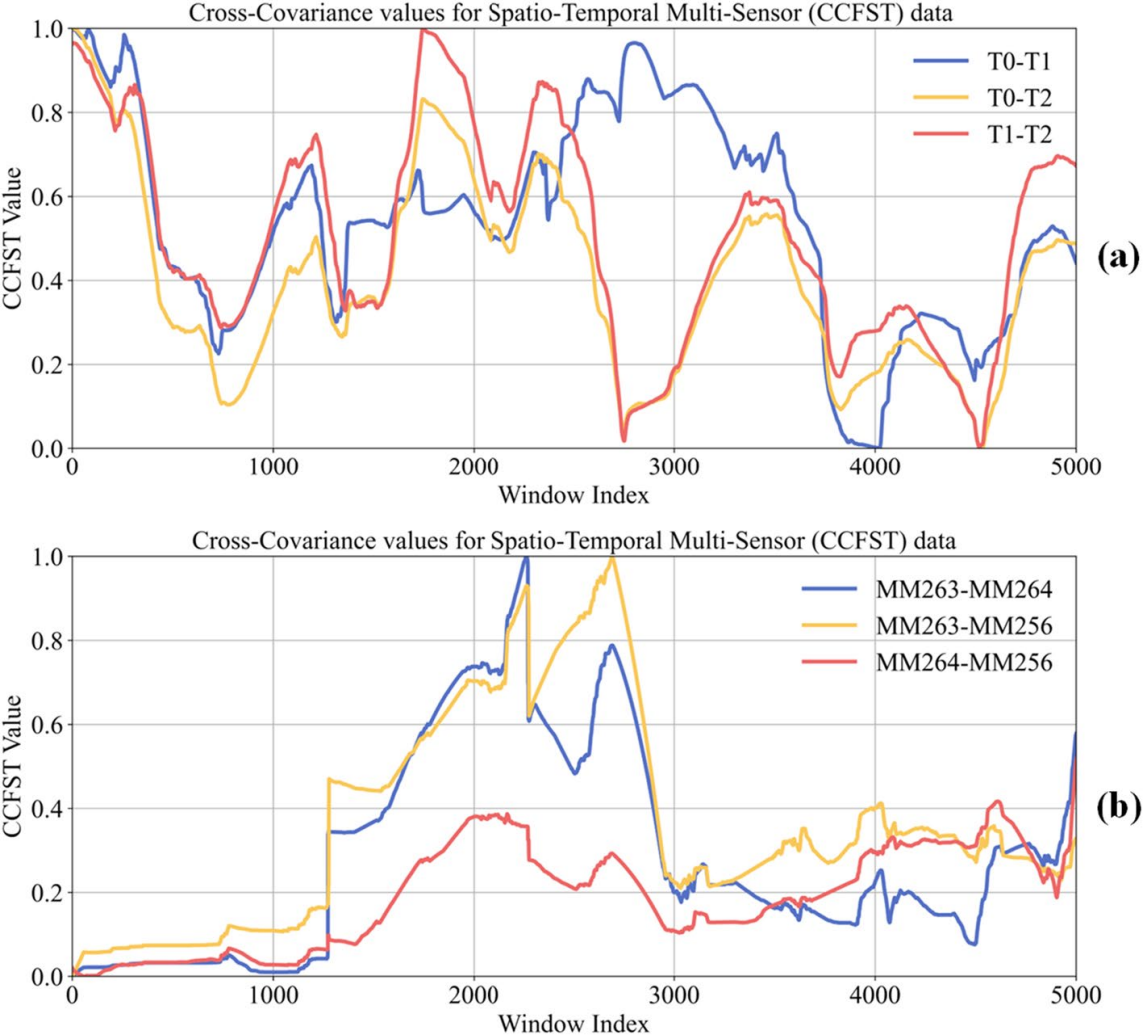
Spatio-temporal correlation between Chinese and Polish coal mines.

To analyze the spatio-temporal correlation within the datasets, we employed a sliding window technique with a window size of 480, equivalent to 8 h of operational activity, applied consistently across all datasets. The computed results are illustrated in Fig. 8a for Chinese coal mines and Fig. 8b for Polish coal mines. Subsequently, the data were normalized to simplify the interpretation, encompassing a total of five thousand sliding windows.

The Cross-correlation function for spatio-temporal data (CCFTS) values indicate the degree of interaction between two points in the dataset. While CCFTS value approaching 1 signifies a strong correlation, indicating that the two points move in a highly synchronous manner. This strong correlation is evident in the consistent trends observed in the data from both Chinese and Polish coal mines throughout the time series. The simultaneous rise and fall in these values across the datasets indicate a persistent underlying pattern, suggesting that similar operational or environmental factors influence both mining locations.

### Model prediction results and risk level determination

**Figure 9:**
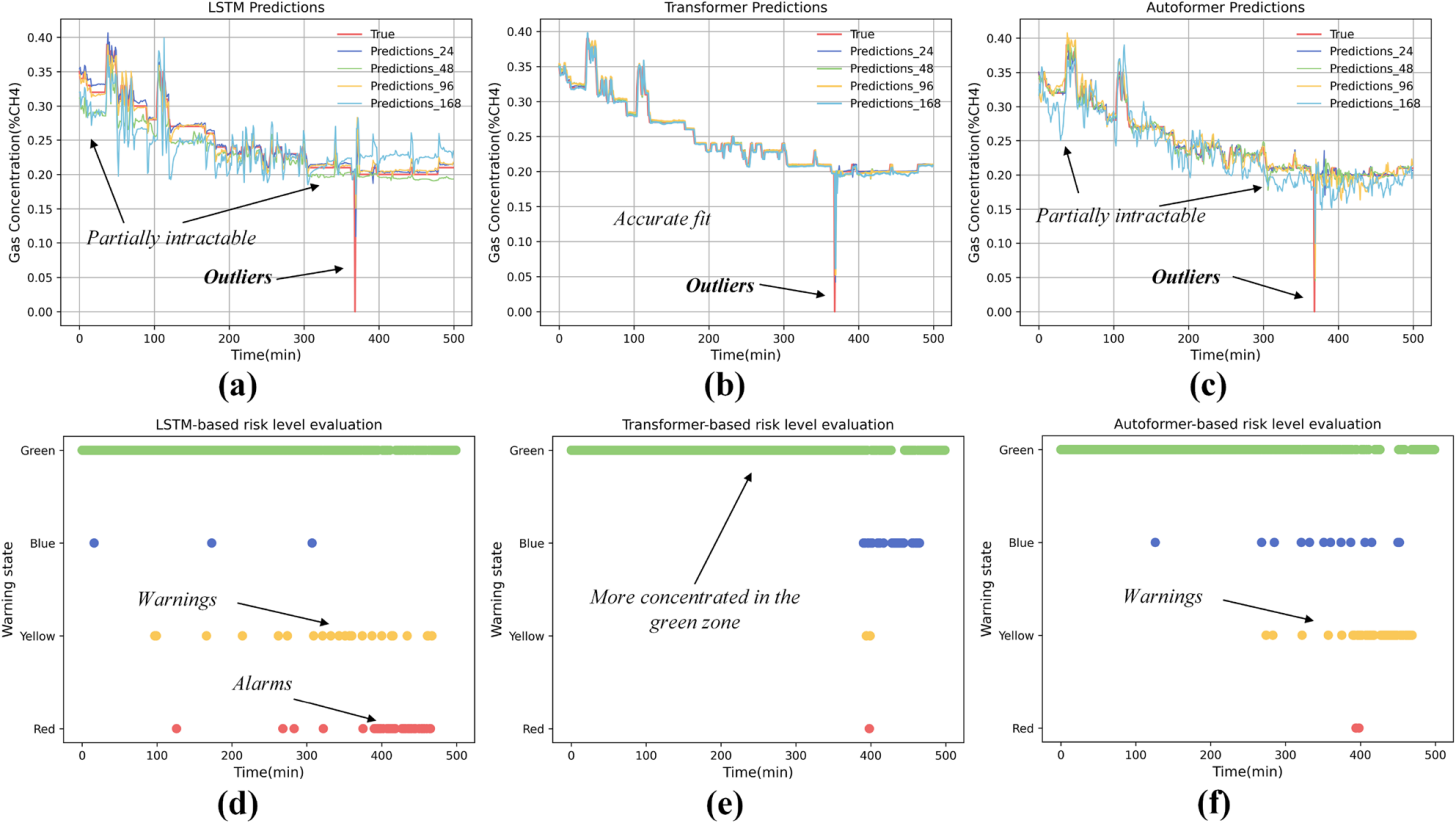
Results of different model predictions and risk level judgements for coal mines in China.

**Figure 10:**
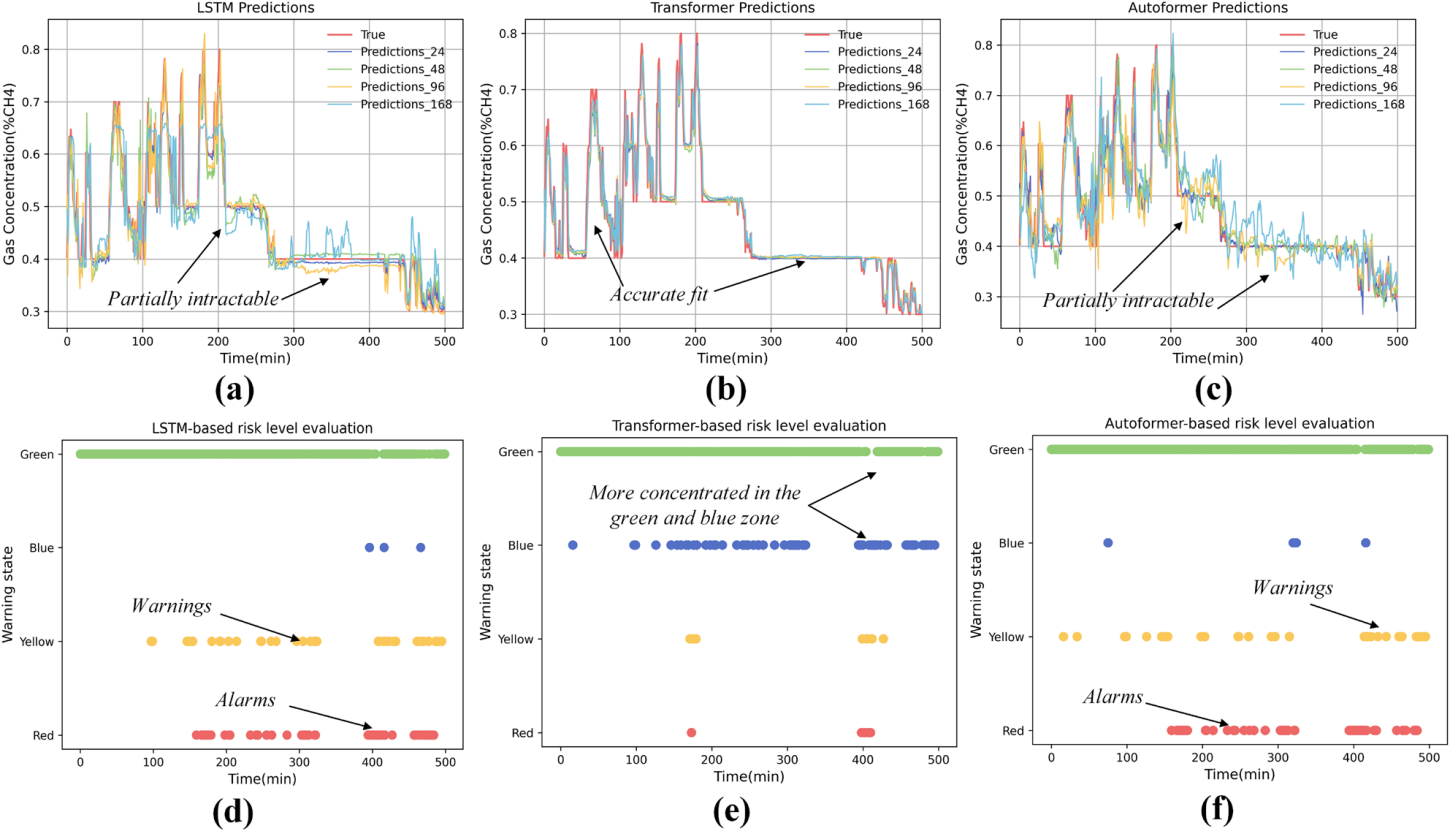
Results of different model predictions and risk level judgements for coal mines in Poland.

We implemented three distinct models to analyze the multivariate time series data across all datasets: LSTM (Long Short-Term Memory)^35^ served as the baseline, with Transformer^36^ and Autoformer^37^ to do multivariate time series prediction, where the position of the upper corner is the predicted value and the others are used as eigenvalues. To verify the effect of different sliding window sizes on the prediction results, we selected [24,48,96,168] sizes for the verification, and this window selection is borrowed from the idea of Autoformer model. At the same time we recorded all the model losses, where the bolded text is the minimum loss, see Table 2. The prediction results for Chinese and Polish coal mines are shown in Figs. 9a–c and 10a–c, respectively. The LSTM model, used here as a baseline, exhibits suboptimal performance in handling multivariate prediction tasks. In contrast, the Autoformer model ranks second, while the Transformer model demonstrates the most robust performance in this scenario. For instance, the LSTM model underperforms compared to the Transformer, particularly in predicting outliers as illustrated in Fig. 9a,c, and also has some challenges with partially intractable.

**Table 2:** Comparison of methods on different metrics for coal mines in China and Poland.

Methods	Metrics	LSTM^35^				Transformer^36^				Autoformer^37^			
		MSE	MAE	RMSE	MAPE	MSE	MAE	RMSE	MAPE	MSE	MAE	RMSE	MAPE
Coal mine in China	24	**0.01**	**0.17**	**0.24**	**0.94**	0.14	0.16	**0.20**	0.92	0.14	**0.15**	**0.21**	**0.78**
	48	**0.01**	0.26	0.31	1.49	**0.01**	0.20	0.25	1.10	**0.01**	0.18	0.24	0.96
	96	0.03	0.45	0.53	2.72	0.11	**0.14**	**0.20**	**0.72**	**0.01**	0.19	0.26	0.99
	168	0.04	0.57	0.65	3.56	0.17	0.17	0.22	0.94	**0.01**	0.19	0.25	1.01
Coal mine in Poland	24	0.43	1.82	2.07	4.24	**0.11**	**0.75**	1.07	**1.56**	**0.02**	**0.27**	**0.46**	**0.61**
	48	0.22	1.18	1.48	2.62	**0.11**	0.76	**1.05**	1.60	**0.02**	0.31	0.49	0.71
	96	**0.19**	**1.02**	**1.39**	**2.13**	0.14	0.86	1.19	1.84	0.03	0.38	0.55	0.88
	168	0.40	1.77	2.01	4.09	0.16	0.93	1.25	1.97	0.03	0.42	0.59	1.00

Significant values are in bold.

The fitting effect of the model is very important for the determination of the risk level of the coal mining face, in Figs. 9d–f and 10d–f the results of the risk level of the Chinese coal mines and the Polish coal mines are shown, respectively, and we opted to display the warning results over a span of 2000 sliding windows, and the size of each window is the same as that in the value of the CCFTS. By predicting the data in each sliding window separately and calculating the value of CCFTS, finally the risk rank is obtained by weighted average. It is interesting to note that as the fit of the model improves, the risk level judged to be red is gradually decreasing, while the risk level in green is gradually increasing, as demonstrated in Fig. 9d–f. This may be due to the fact that the model is a poor predictor and gets a large loss, this loss exceeds the loss threshold while at this time the value of the CCFTS, which is the distance between the sensors, remains constant, thus ending up with a higher risk rating. As the accuracy of the model improves, the loss decreases and the majority of the risk level is judged to be green.

### Discussions

Despite evidence of similar spatial and temporal correlations and model generalization across different mines, choosing the appropriate parameters, such as the size of the time window, becomes crucial. Factors like underground wind speed and the structure of the coal mining face are essential in determining more precise time lags for the results. Furthermore, models based on time series prediction significantly influence the risk rating outcomes, as evidenced by the data shown in Figs. 9 and 10. Challenges persist, particularly as patterns in industrial data evolve over time, such as coal mining and tunneling, the relocation of sensors as the excavation progresses poses additional complexities. Additionally, validating the timeliness of the data remains a critical area for future research. Considering the opaque nature of deep learning models, future studies focusing on interpretable AI may offer a more promising direction, aiming to provide clearer insights into the decision-making processes of these models.

## Conclusion

Intelligent computing is transforming coal mine safety through advanced gas risk assessments and enhanced countermeasure planning. This study introduces a predictive model that utilizes multi-source data to assess gas risk levels effectively, refining responses by examining gas drift patterns in longwall mining using temporal and spatial correlations. Supported by a four-tier early warning system, the model integrates weighted predictive confidence with extensive data analysis. Initially tested using a public dataset in Poland and later validated in a Chinese coal mine, the method has demonstrated some effectiveness in real-world scenarios. This underscores the value of multi-source data in enhancing correlation analyses and developing a robust risk warning mechanism that improves safety and regulatory management within the mining industry. Although current results are promising, the complexity and variability of underground environments call for extensive future field testing. Pursuing deep learning research that includes more variables, or achieving comprehensive environmental awareness across the entire working face, remains an exciting future research direction.

## Data Availability

The data used in this paper are available on request from the corresponding author.
